# Stress-Strain Behavior of Cementitious Materials with Different Sizes

**DOI:** 10.1155/2014/919154

**Published:** 2014-03-13

**Authors:** Jikai Zhou, Pingping Qian, Xudong Chen

**Affiliations:** College of Civil and Transportation Engineering, Hohai University, Nanjing 210098, China

## Abstract

The size dependence of flexural properties of cement mortar and concrete beams is investigated. Bazant's size effect law and modified size effect law by Kim and Eo give a very good fit to the flexural strength of both cement mortar and concrete. As observed in the test results, a strong size effect in flexural strength is found in cement mortar than in concrete. A modification has been suggested to Li's equation for describing the stress-strain curve of cement mortar and concrete by incorporating two different correction factors, the factors contained in the modified equation being established empirically as a function of specimen size. A comparison of the predictions of this equation with test data generated in this study shows good agreement.

## 1. Introduction

It is widely believed that the true fracture properties of concrete structures can be unequivocally determined only by means of uniaxial tensile stress [1]. Unfortunately, tensile tests are difficult to carry out in standard laboratories, either with fixed or rotating boundary conditions. Therefore, the flexure strength, measured for beams in either three- or four-point bending, turns out to be an experimentally convenient measure of strength owing to the relative simplicity of these results [2–9].

It is important to consider the effect of size while estimating the ultimate strength of a concrete member under various loading conditions. Presently, most design codes for concrete structures do not consider the effect of size. Since quasibrittle materials fail by formation of cracks, size effect has to be implemented. The influence of specimen diameter [10, 11] and slenderness ratio [12–14] on the compressive strength of concrete has been reported by many investigators. Less is known about the size effects when passing from the uniaxial tension domain into the flexural tension range. Four-point bending test was analyzed theoretically by Ghaemmaghami and Ghaemian [15] using a cohesive crack model. The results obtained with the model showed that the flexural strength of quasibrittle materials such as concrete, ceramics, and rocks is a decreasing function of the specimen size, whose value decreases as the size increases. Vořechovský [16] also noted that the tensile strength was different depending on the type of loading applied to specimens of the same volume and geometry. Experiments show that the average tensile strength computed from four-point bending beams are increased by 200 percent and by 220 percent in three-point bending from what is measured in direct tension. Experiments on notched specimens of homothetic sizes made of concrete, rocks, and ceramics by Bazant et al. [17, 18] and Man and Van Mier [19] also demonstrate that the nominal tensile and shear strengths depend on the size of the specimens. All these experiments show two key points: (1) the flexural strength of cement-based materials as predicted by elasticity or limit analysis is a function of the volume of the specimen and of the stress field in the structure. Part of this size effect can be attributed to the existence of initial defects in the material before any stress is applied to it, and (2) the fracture toughness measure from notched specimens also varies with the size of the structure. Failure in these materials is often the result of progressive microcracking in a fracture process zone whose size is related to the size of inhomogeneities or aggregates. From the literature it can be found that there are many studies on mechanical characteristics of notched beams and only a few on the notchless beams. The present study is aimed at documenting the effects of size in notchless cement mortar and concrete beams.

The size effects on the strength of concrete materials have been generally explained as a macroscopic phenomenon resulting from internal microscopic defects such as microcracks and inclusions in the material. Mazars et al. [20], Kameswara Rao and Swamy [21], and Kumar and Barai [22] have tried to apply some theoretical approaches to clarify the size effect on the strength of concrete. Most of the researchers have set up the theories based on the assumption that the size effect in strength is related to the quantity of defects in the specimen, that is, the size of specimen. Intensive studies on the strength of concrete in the last decades have clarified that the size effect on strength is related to primarily to a relatively large fracture propagation process (FPZ) in concrete [23]. Nonlinear fracture models, that is, the Fictitious Crack Model by Hillerborg et al. [24], the Crack Band Model by Bazant [25], and the Two-Parameter Model by Jenq and Shah [26], have been applied to the analysis and prediction of size effects in concrete strength. Successively, Carpinteri et al. [27] proposed the Multi-Fractal Scaling Law (MFSL), valid for initially integer specimens and components. However, such investigations have often been restricted to strength and the deformation has not been reported.

Knowledge of the stress-strain relationship of concrete is vital to achieve a rational design of structures and structure components involving concrete. The nonlinear stress-strain behavior, size-dependent strength, fracture mode transition, and other phenomena are not adequately explained by the classical concepts of the mechanical behavior of materials and mechanics of fracture [28–30]. Enquiry into such aspects of the behavior of materials is essential in assessing the material properties of engineering importance, correlating the observations from tests on modes to those of prototypes, and so forth. With regard to the stress-strain relationship, studies reporting the size effect are very scarce.

In this paper, the authors discuss the size effects on cement mortar and concrete flexural behavior quantitatively. The equations to estimate stress-strain curves were also proposed. Results of the investigation on the validity of the existing models are reported as well. This paper brings together the results of past studies and the results of new experiments and interpretations.

## 2. Experimental Program

### 2.1. Materials and Specimens

Ordinary Portland cement (OPC) was used in the production of cement mortar specimens. The cement was the most widely used cement in general concrete construction works in China. The fine aggregate was river sand consisting mainly of quartz, with 10 percent feldspar. The gradation test showed that the particle size of the sand was continuously distributed within the range of 0.4–2.5 mm with 80% of sand. The water-cement ratio (*w*/*c*) 0.4 and sand-cement ratio (*s*/*c*) 2 were used for cement mortar. The cement mortar also has the same compositions as the mortar phase in the concrete. When tested separately, it provides a mean for obtaining the response of the mortar in concrete. It must be pointed out that the* in situ* properties of mortar phase may not exactly the same as those of the pure mortar; especially the degree of porosity in the matrix could be different from that of the pure mortar [31]. The crushed granite was used as the aggregate of concrete and its maximum grain size was 15 mm. The aggregate-cement ratio (*a*/*c*) 1.5 was used for concrete.

**Figure 1:**
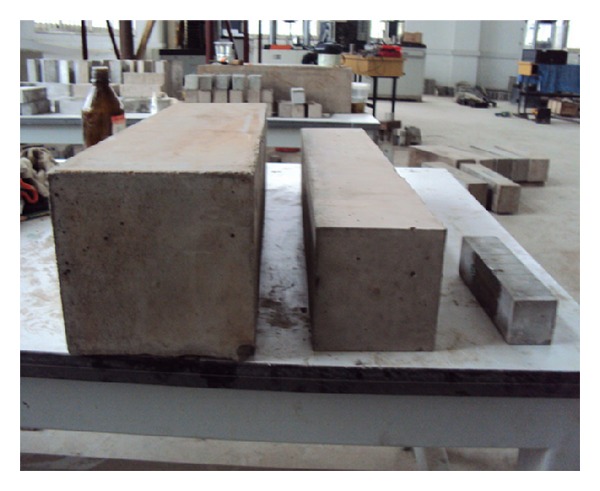
Concrete specimens with beam depths of 40, 100, and 150 mm.

Test specimens of three different sizes were cast. Their dimensions were (width × depth × length): (1)  40 × 40 × 160 mm, tested on a 120 mm span; (2)100 × 100 × 400 mm, tested on a 300 mm span; and (3)150 × 150 × 550 mm, tested on a 450 mm span.  Figure 1 shows the three different size concrete specimens used in this investigation. Three specimens of each size were cast. The forms were covered with wet burlap for the first two days; the specimens were then cured in the ambient laboratory environment for a further 34 to 36 days until they were tested. Three companion 150 cubes were also cast and subjected to the same curing. They were tested at an age of 38 days, yielding an average compressive strength of 52.3 MPa for cement mortar and 65.61 MPa for concrete.

### 2.2. Four-Point Loading Tests

**Figure 2:**
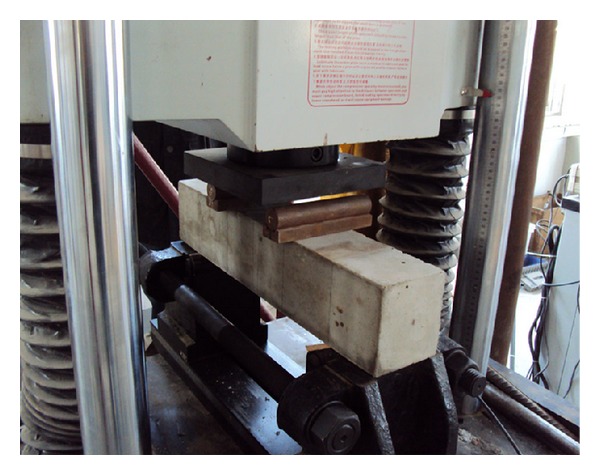
Picture of test setup for four-point loading.

**Figure 3:**
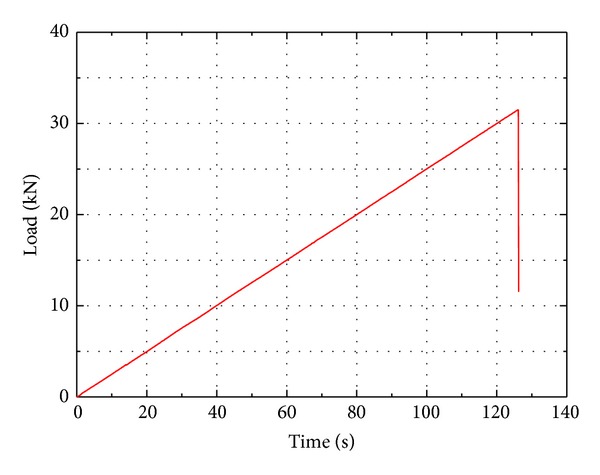
Typical load time curves for concrete specimen.

**Figure 4:**
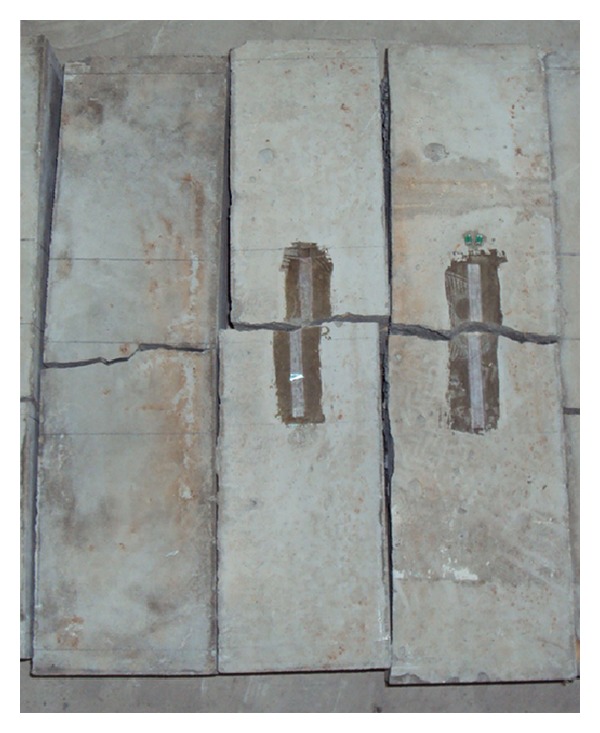
Failure mode of concrete specimens after flexural tests.

As aforementioned, in order to study the influence of specimen size on flexural properties of cement mortar and concrete, four-point bending tests on beams were conducted. The testing was conducted using a dynamic loading system that applied a programmable controlled loading. All these tests were performed with a closed loop servo-controlled stiff testing machine. The load was applied at an approximately constant deformation rate of 0.25 kN/s. The test setup is shown in Figure 2. The width of the support was kept constant as it was not thought that this would have any influence on the results. The measurement system consists of a strain amplifier, a tape recorder, and an intelligent signal processor. A 10^4^ Hz sampling frequency can be achieved. Eleven pairs of foil strain gages, 5 mm in width and 50 mm in width, were used to monitor the strain histories. They were crossly glued onto the faces of the specimen. Each specimen was removed from the moist room 2 days before testing. Typical load-time curve for a concrete beam test at the loading is shown in Figure 3. This illustrates that the linearity of loading was acceptable and that failure was easily recognized.  Figure 4 shows the typical flexural failure mode of concrete beams; the failure location of all concrete beams is at the midspan.

## 3. Test Results and Discussion

The discussion of the results is organized as follows. First we present and describe the strength results obtained from the tests. Then we proceed to discuss the size effect in stress-strain curves. In addition, the test results were compared with existing scaling laws.

### 3.1. Size Effect on Strength

**Figure 5:**
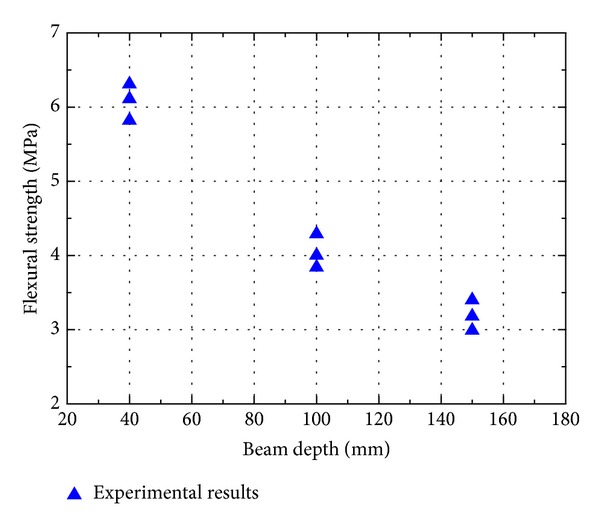
Influence of beam size on flexural strength of cement mortar.

**Figure 6:**
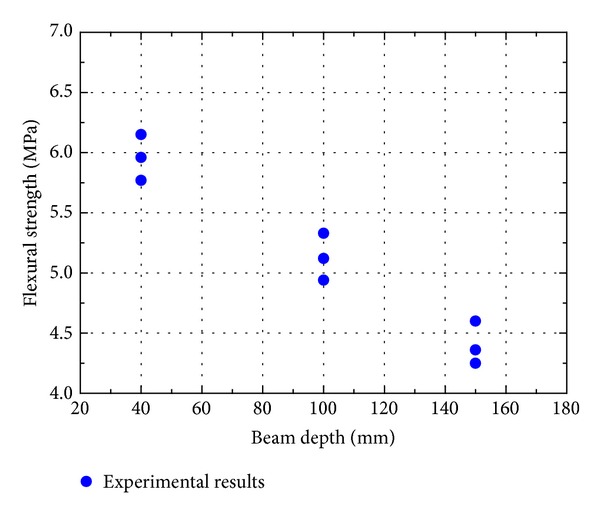
Influence of beam size on flexural strength of concrete.

The flexural strength of cement mortar and concrete measured from different sizes of beams are shown in Figures 5 and 6. The flexural strength of both cement mortar and concrete decreases with the specimen size increases, which is similar to the trend observed by others [23, 27, 29, 32]. But a strong size effect in flexural strength is found in cement mortar than in concrete. Kani [33] was one of the first to demonstrate the size effect in concrete structures. It has been shown that the strength of similar concrete beams decreases with increasing beam depth. Due to the fracture in a structural element being driven by storing energy released from the whole structure, this size effect can be well interpreted by fracture mechanics. The fact that the strength of brittle materials is affected by the presence of defects is first suggested by Griffith [34]. Due to his conclusion, it can be expected that the value of the ultimate strength will depend upon the size of specimen. As specimen size increases, the strength is expected to be decreased since the probability of presence of weak links is increasing. Traditionally, the size effect in fracture of concrete structural elements has been explained as Weibull's theory [35]. He showed that if tensile tests are performed on two geometrically similar specimens with different volumes, the corresponding ultimate strengths are different. It has also been concluded by other researchers [36–40].

Glucklich and Cohen [41] attribute the decrease in the failure strength with increased size to a strain energy mechanism. They describe the mechanism of stable crack growth based on the elastic energy stored within a specimen, with the amount of energy stored being correlative with specimen size. A sudden drop in the resistance of the specimen is followed by a decrease in the driving force; the energy rate exceeds that of energy demand; equilibrium breaks down; and accelerated failure takes place. When a system is overstocked with strain energy due, say, to the largeness of the specimen, any sudden drop in energy demand creates an excess of energy released. This then takes the form of kinetic energy which then advances fracture, favoring the conditions of unstable crack propagation. This mechanism may be plausible in the case of concrete materials, because Glucklich and Cohen [41] do not account for the constant maximum strength values for specimen above a certain size.

The other explanation of the size effect on flexural strength is to be found in the theory of quasibrittle fracture, describing materials of heterogeneous microstructure in which the formation of distinct fractures is preceded by distributed cracking. The failure of a beam begins by distributed cracking that develops in a boundary layer. The thickness of this layer for different beam sizes is about the same, provided the same concrete is considered. Hillerborg et al. [24] showed by numerical calculations that the stress distribution at the peak load has a maximum that lies at a certain distance from the tensile face. This distance is determined by the softening stress-displacement relation to the cohesive (fictitious) crack model. Dyskin et al. [42] and Karihaloo et al. [43] also demonstrated that numerical calculations based on the cohesive model can match the test results on the influence of beam size.

Some relationships involving strength and size of engineering materials have been reported in the literature. Historically, several general types of model have been developed for cement-based materials. As stated above, the aim of the investigation is to judge the range of applicability of the various size effect formulae available in the literature.

**Figure 7:**
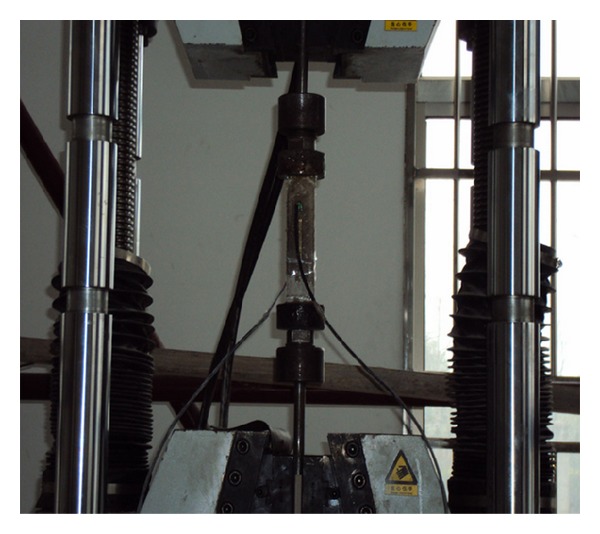
Picture of test setup for direct tension test.

On the basis of the experimental results, an empirical expression was proposed by Xu and He [44], relating the nominal flexural strength *σ*_*u*_ to the depth of *d* of the specimens:

(1) $$\begin{array}{c} \sigma_{u}=\left(0.8+0.26\cdot d^{-0.6}\right)f_{t}, \end{array}$$

where *f*_*t*_ is the uniaxial tensile strength, which in our cases we take as the strength that is obtained from the direct tension test in this investigation (as shown in Figure 7). Note that, according to (1), the flexural strength of very deep beams (*d* > 1500 mm) becomes smaller than *σ*_0_. Note also that these arguments would imply that the nominal flexural strength increases if applied compressive loads act upon the beam, thus reducing the size of the tension zone.

Based on a similar strain-gradient approach, a size dependent empirical relationship for the flexural strength has been specified in the CEB-FIP Model Code 1990 [45] and is given by

(2) $$\begin{array}{c} \sigma_{u}=f_{t}\left[\frac{1+2.0{\left(d/d_{0}\right)}^{0.7}}{2.0{\left(d/d_{0}\right)}^{0.7}}\right], \end{array}$$

where *d*_0_ is a reference size equal to 100 mm. Note that (2) comes from experimental observations and no consideration is given to the role of the microstructure, whereas aggregate interlocking is explicitly taken into account in the codes when dealing with the ultimate shear strength.

In the analysis of the flexural strength of concrete, Rokugo et al. [46] employed a finite element method incorporating tension softening. It was found that the flexural strength can be expressed as a function of the ratio of the beam depth to the characteristic length. Based on the results, the authors proposed an equation to estimating the flexural strength of specimens with different sizes as follows:

(3) $$\begin{array}{c} \sigma_{u}=f_{t}\left[1+\frac{1}{0.85+4.5\left(d/l_{H}\right)}\right], \end{array}$$

where *l*_*H*_ is Hillerborg's characteristic length [24] and *d*/*l*_*H*_ ≥ 0.1. For cement-based materials, *l*_*H*_ = 300 mm.

According to Bazant [25], the size effect in solids is a smooth transition from the strength criterion of plasticity (applicable to small size specimens) to the crack size dependence of linear elastic fracture mechanics (LEFM) (as seen in larger specimens). The failure stress of s series of geometrically similar specimens of concrete is described by the following infinite series:

(4) $$\begin{array}{c} \sigma_{u}=Bf_{t}{\left(\frac{d}{d_{0}}+1+{\left(\frac{d}{d_{0}}\right)}^{-1}A_{1}+{\left(\frac{d}{d_{0}}\right)}^{-2}A_{2}+\cdots \right)}^{-1/2}, \end{array}$$

where *B*, *d*_0_, and *A*_*i*_ (*i* ∈ [1, *∞*]) are empirical constants obtained from experimental data. For the size rang up to *d*/*d*_0_ ≤ 1/20, the infinite series, (4), may be truncated to the first two terms. Thus, Bazant's size effect law (BSEL) reduces to

(5) $$\begin{array}{c} \sigma_{u}=Bf_{t}{\left(1+\frac{d}{d_{0}}\right)}^{-1/2}. \end{array}$$

In fact, (5) has been established by Taylor's expansion from this asymptotic limit [25]. The positive coefficients *B* and *d*_0_ in (5) are related to the specific fracture energy and the fracture process zone (FPZ) size of a very large structure (*d* → *∞*), as well as to the nondimensional geometry factor and its first derivative. The geometry factor depends on the notch to depth ratio and the geometry of the test specimen.

By considering a constant maximum flaw size, as is likely to occur in real materials, Kim and Eo [47] proposed the modified size effect law by adding to the BSEL the size-independent strength *σ*_0_ (=*αf*_*t*_):

(6) $$\begin{array}{c} \sigma_{u}=Bf_{t}{\left(1+\frac{d}{d_{0}}\right)}^{-1/2}+0.15f_{t}. \end{array}$$

Chen et al. [9] used the concept of self-similar morphologies with noninteger dimensions called fractals to describe the microstructure of quasibrittle materials such as cement-based materials. With an increase in the scale of observation, the topological fractality is thought to vanish. On the bases of this hypothesis, the following multifractal scaling law (MFSL) was proposed:

(7) $$\begin{array}{c} \sigma_{u}=f_{t}{\left(1+\frac{l_{\text{ch}}}{d}\right)}^{1/2}, \end{array}$$

where *l*_ch_ is the characteristic length of the material.

Karihaloo et al. [48], using the stress intensity factor (SIF) and the fictitious crack concepts, proposed the formula:

(8) $$\begin{array}{c} \sigma_{u}=f_{t}\left(1+\frac{2C}{d}\right), \end{array}$$

where *C* is constant. This formula is, however, unlikely to be applicable when *d* is small, which is a consequence of several approximations and assumptions made in its derivation. The constant *C* in (8) is also related to the FPZ size of a very large structure and to the nondimensional geometry and its first derivative.

**Figure 8:**
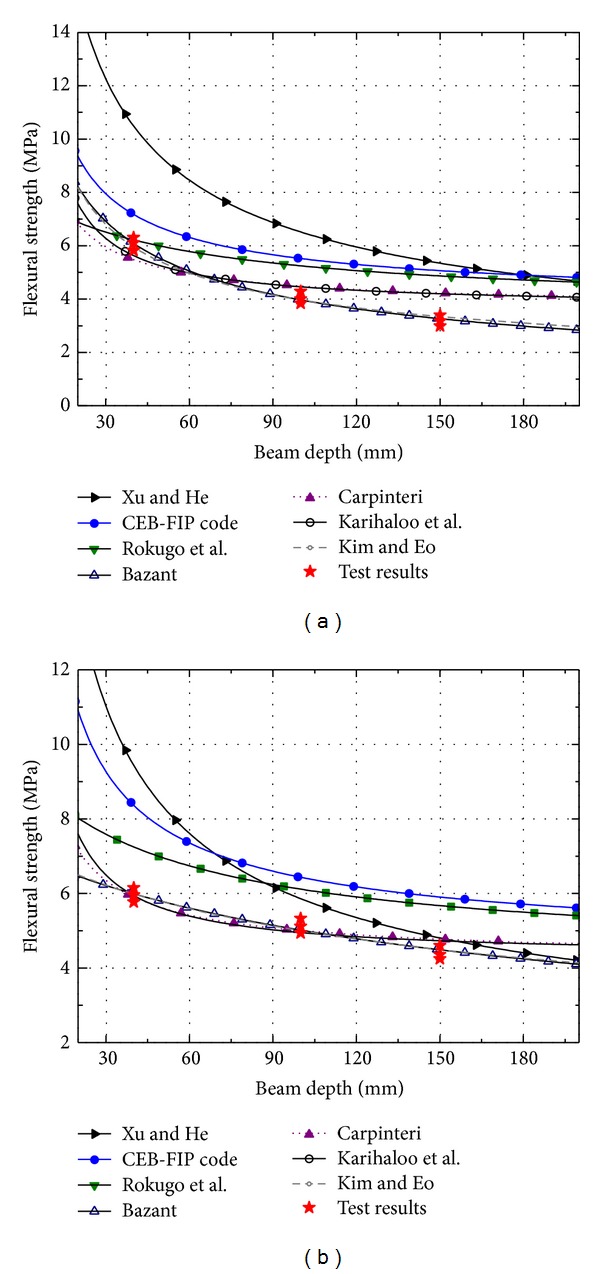
Comparison of test data with existing scaling laws: (a) cement mortar; (b) concrete.

By plotting these relationships against the experimental results in this study (Figure 8), the correspondence with the data obtained can then be appreciated. From literature review and Figure 8, it can be found that the empirical model agrees well with their respective experimental data and less well with the experimental data obtained by other authors, because each proposed equation was obtained by using a regression analysis to interpolate their own experimental data. Moreover, appreciable difference arises between the curves, either in the case of the smaller, or, which is more important, in the case of larger beam sizes (Figure 8). Note that Bazant's size effect law and the modified size effect law proposed by Kim and Eo [47] are in agreement with the test results of this investigation. The same results have also obtained by other researchers [32, 49].

### 3.2. Stress-Strain Curves

**Figure 9:**
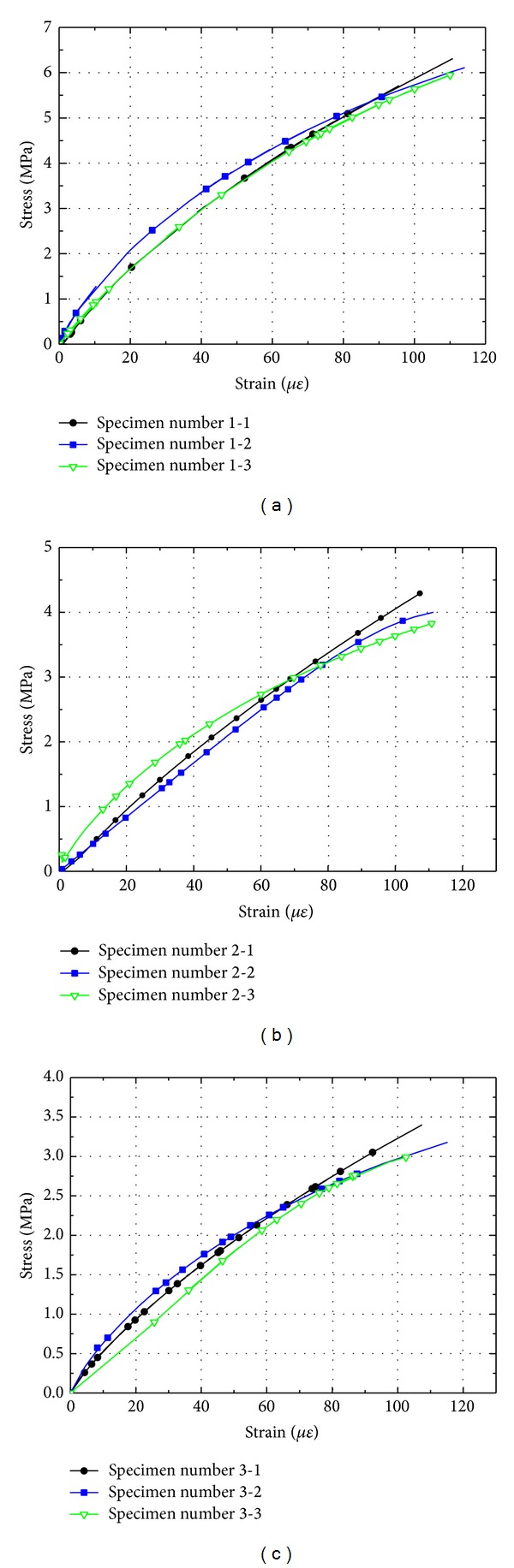
Influence of beam size on stress-strain curves of cement mortar: (a) beam depth 40 mm; (b) beam depth 100 mm; (c) beam depth 150 mm.

**Figure 10:**
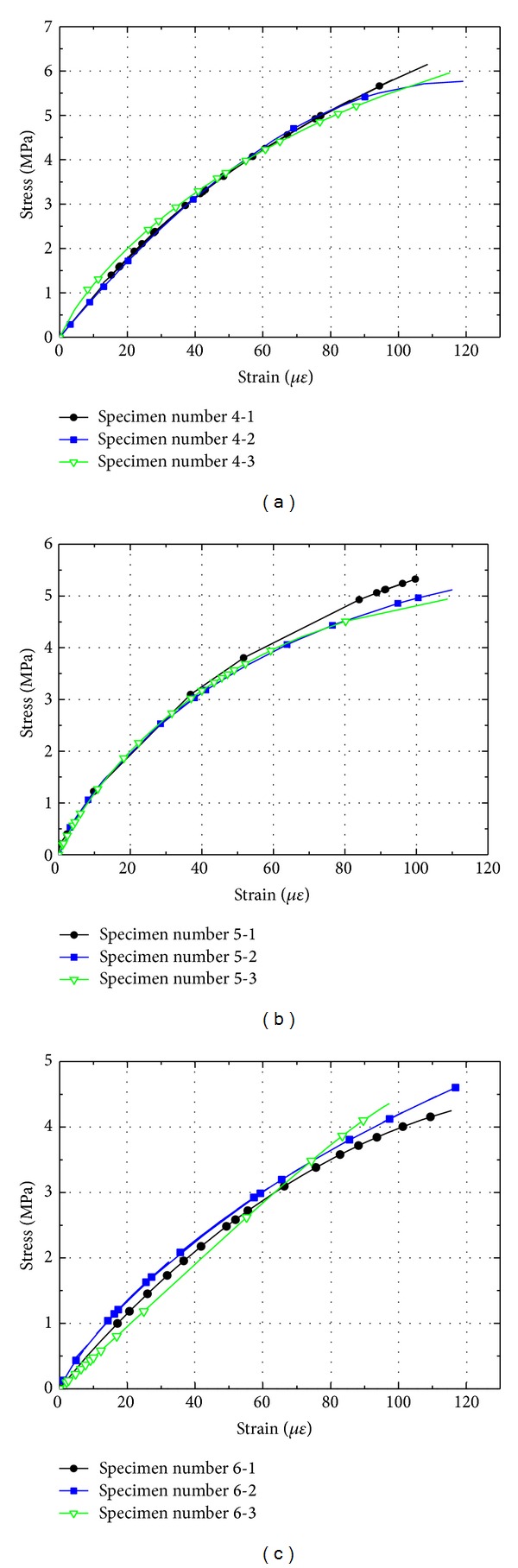
Influence of beam size on stress-strain curves of concrete: (a) beam depth 40 mm; (b) beam depth 100 mm; (c) beam depth 150 mm.

The stress-strain curves of cement mortar and concrete with different sizes are shown in Figures 9 and 10. It is seen that the stress-strain curves of the three sizes of specimens behave considerably different. It is evident from Figures 9 and 10 that the critical strain at the peak load is more or less constant and independent of specimen size. This indicates that a major source for the size effect of strength for cement-based materials may actually be the material's modulus of elasticity. Strength being a macroscopic measure for the material is probably influenced by the fluctuation of the modulus of elasticity, which in turn is influenced by the aggregate size to specimen size ratio [12, 16]. Microscopically, the cement-based material of each mix is identical, independent of specimen size. Thus, the characteristic strain at peak load should also be independent of specimen size, as it appears to be. The strain invariance in combination with the variance of the modulus of elasticity with specimen size appears to be a possible explanation of the so-called size effect of material strength.

## 4. Modeling Stress-Strain Relationship Based on Continuous Damage Mechanics

Damage mechanics has become a powerful tool for the modeling of the nonlinear behavior of materials subjected to a progressive microcracking process. The theory itself was initially developed in 1958 [50], and, in the particular case of concrete, different constitutive models based on scalar, tensorial damage concepts have been formulated since then [51–53].

Among many types of concrete damage models at hand, one established model [54] was taken, where the damage variable *D* is defined as

(9) $$\begin{array}{c} D=1-\frac{\tilde{A}}{A} \end{array}$$

in which $\tilde{A}$ is the area of the cross section with damage and *A* is the area without damage. Moreover, the effective stress *σ*_*e*_ is defined as follows:

(10) $$\begin{array}{c} \sigma_{e}=\frac{\sigma }{1-D}, \end{array}$$

where *σ* is the macroscopic stress.

According to the equivalent strain principle in damage mechanics, the damage constitutive relationship of concrete was established by Li et al. [54] as

(11) $$\begin{array}{c} \sigma =E\varepsilon \left(1-D\right),\\ D={\left(\frac{\varepsilon -\varepsilon_{0}}{k}\right)}^{n}, \end{array}$$

where *ε*_0_ is threshold strain, below which no damage occurs; *E* is the elastic modulus (GPa); and *k*, *n* are damage parameters of the stress-strain curve.

The effect of specimen size was taken into account by assuming that the threshold strain (*ε*_0_, 10^−6^), and the elastic modulus (*E*, GPa) are size-dependent. For cement mortar, *n* = 0.47, and *k* = 161 × 10^−6^; for concrete, *n* = 0.83, and *k* = 223 × 10^−6^. The following relationships were derived from the experimental results:

 for cement mortar,
  (12) $$\begin{array}{c} E=30.0\cdot {\left(\frac{d}{575.5}\right)}^{-0.16},\\ \varepsilon_{0}=95.7-598.1\cdot {\left(\frac{807}{d}\right)}^{-1.94}; \end{array}$$
 for concrete,
  (13) $$\begin{array}{c} E=31.0\cdot {\left(\frac{d}{575.5}\right)}^{-0.27},\\ \varepsilon_{0}=94.5-728.2\cdot {\left(\frac{807}{d}\right)}^{-2.36}. \end{array}$$

**Figure 11:**
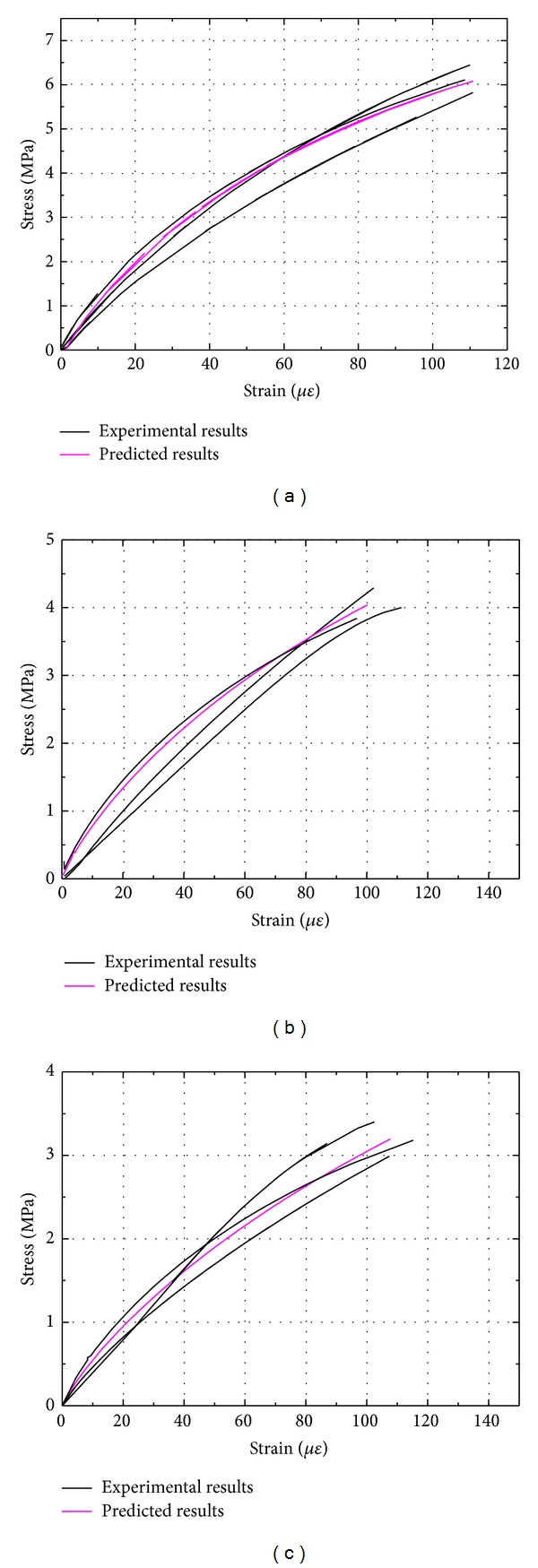
Comparison of predicted results with test data for cement mortar: (a) beam depth 40 mm; (b) beam depth 100 mm; (c) beam depth 150 mm.

**Figure 12:**
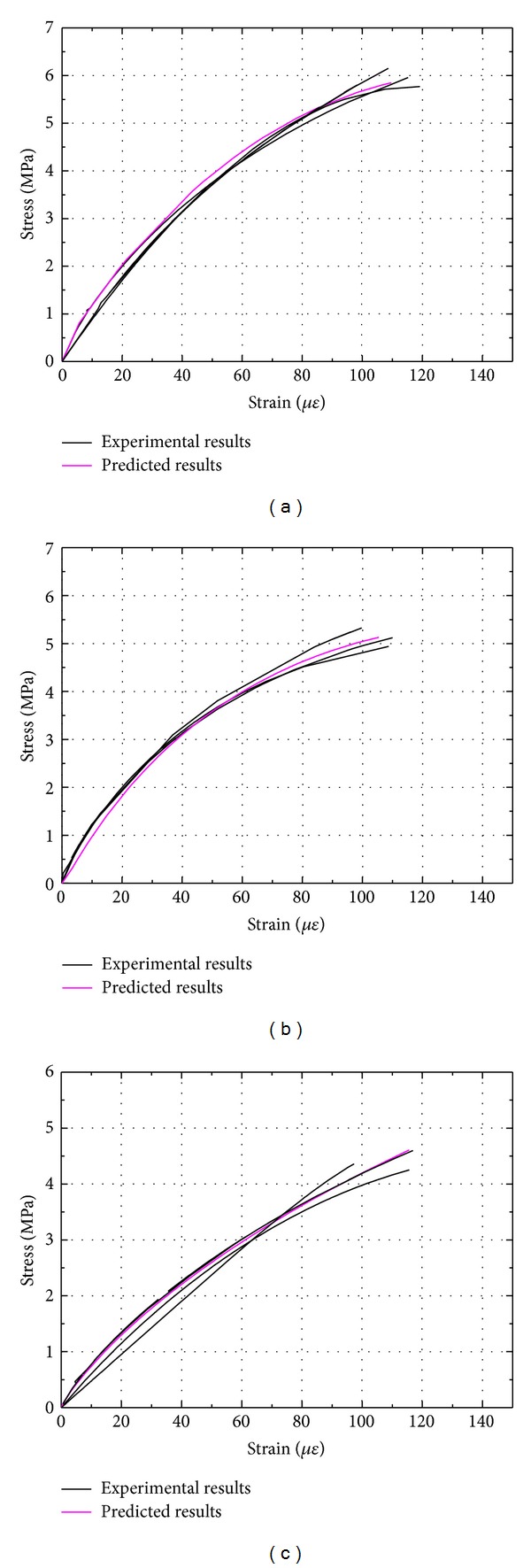
Comparison of predicted results with test data for concrete: (a) beam depth 40 mm; (b) beam depth 100 mm; (c) beam depth 150 mm.

The predictions of the analytical model thus computed are compared with the experimental data for different sizes of cement mortar and concrete in Figures 11 and 12. It may be seen that the theoretical predictions are in good agreement with the experimental curves.

## 5. Conclusions

In this study, the flexural behavior of cement mortar and concrete with different sizes were investigated. From the test results, the following conclusions can be drawn.

1. Test results of flexural strength show size effect. Large specimens resist less in terms of stress than the smaller ones. A strong size effect in flexural strength is found in cement mortar than in concrete. The size effect in flexural strength is compared with existing scaling laws; it is found that Bazant's size effect law and the modified size effect law proposed by Kim and Eo are in agreement with the test results.
2. A modification has been suggested to Li's equation for describing the stress-strain curve of cement mortar and concrete by incorporating two different correction factors, the factors contained in the modified equation being established empirically as a function of specimen size. A comparison of the predictions of this equation with test data generated in this study shows good agreement.
